# Improvement of Detection Sensitivity of Microbubbles as Sensors to Detect Ambient Pressure †

**DOI:** 10.3390/s18124083

**Published:** 2018-11-22

**Authors:** Fei Li, Deyu Li, Fei Yan

**Affiliations:** 1 Paul C. Lauterbur Research Center for Biomedical Imaging, Shenzhen Institutes of Advanced Technology, Chinese Academy of Sciences, Shenzhen 518055, China; fei.li@siat.ac.cn; 2 Shenzhen Key Laboratory of Ultrasound Imaging and Therapy, Shenzhen 518055, China; 3 School of Biological Science and Medical Engineering, Beihang University, Beijing 100083, China

**Keywords:** microbubbles, pressure sensors, noninvasive blood pressure measurement, mechanical index, subharmonic amplitude

## Abstract

Microbubbles are considered a promising tool for noninvasive estimation of local blood pressure. It is reported that the subharmonic scattering amplitude of microbubbles decreases by 9 to 12 dB when immersed in the media under an ambient pressure variation from 0 to 180 mmHg. However, the pressure sensitivity still needs to be improved to satisfy clinical diagnostic requirements. Here, we investigated the effects of acoustic parameters on the pressure sensitivity of microbubbles through measuring the acoustic attenuation and scattering properties of commercially available SonoVue microbubbles. Our results showed that the first harmonic, subharmonic, and ultraharmonic amplitudes of microbubbles were reduced by 6.6 dB, 10.9 dB, and 9.3 dB at 0.225 mechanical index (MI), 4.6 dB, 19.8 dB, and 12.3 dB at 0.25 MI, and 18.5 dB, 17.6 dB, and 12.6 dB at 0.3 MI, respectively, when the ambient pressure increased from 0 to 180 mmHg. Our finding revealed that a moderate MI (0.25–0.4) exciting microbubbles could significantly improve their sensitivities to detect ambient pressure.

## 1. Introduction

Measuring hydrostatic pressures in heart cavities and major vessels would provide clinicians with important information for evaluating valvular heart disease, congestive heart failure, portal and pulmonary hypertension, and various vascular diseases [1]. Conventional clinical local blood pressure measurement is invasive through inserting one or several pressure catheters into heart cavities and large blood vessels to obtain the blood pressure, occasionally resulting in increased pain and infection risk for the patients [2]. In addition, the inserted plastic tube can also change the hemodynamics of the surrounding medium, leading to an inaccurate measurement of blood pressure [3]. The ultrasound contrast agent (UCA) microbubble-based sensor is a promising noninvasive approach for blood pressure measurements [4]. Because the acoustic characteristics of these gas-filled bubbles correlate well with the local ambient pressure, they may function as pressure sensors under appropriate ultrasound excitation. As a result, changes in the local blood pressure can lead to variation in the acoustic characteristics of microbubbles. 

To date, there are numerous reports about microbubbles as sensors for the local blood pressure measurements through detecting disappearance time of free bubbles [5,6], shift of the first harmonic or subharmonic resonance frequency [4,7,8,9,10,11,12], or amplitude variation of the scattered echo of microbubbles [1,3,10,13,14,15,16,17,18,19,20,21,22,23,24,25,26,27,28,29,30]. Among them, amplitude variation of the scattered echo is most attractable and promising for clinical application. For instance, Hӧk suggested that blood pressure can be estimated using an echo amplitude from a single bubble [13]. However, the variations of the echo amplitude was very difficult to measure precisely. *In vitro* measurements usually had errors exceeding 30% (<24 mmHg) [5]. Shi et al. found that the subharmonic amplitude of Levovist had a reduction of 9.6 dB in a 0–186 mmHg pressure range, with an ambient pressure sensitivity of −19.38 mmHg/dB. The reduction in subharmonic amplitude was larger than the amplitude decreases of the first and second harmonics (<3 dB) [1]. An excellent linear correlation existed between the subharmonic amplitude and ambient pressure. The relationship between the subharmonic component and ambient pressure was also validated in other several commercially available UCAs, including Optison, Sonazoid, and SonoVue etc. The literature reveals that for these UCAs there were 9–12 dB subharmonic amplitude reductions around 180 mmHg [3,20,21,22,23,24].

Based on the above results, the technique called the subharmonic-aided pressure estimation (SHAPE) for noninvasive blood pressure estimation was proposed and proven in the animal and human experiments [19,31,32,33]. For animal experiments, the results from the SHAPE method were consistent with the data from catheter, with <2.5 mmHg pressure errors [19]. The feasibility of SHAPE was further evaluated in left and right ventricles of humans [2]. In a clinical trial with 45 patients, SHAPE measurements correlated well with transjugular hepatic venous pressure gradient measurements and was potentially used to estimate portal hypertension [34]. All of these investigations demonstrated that the local blood pressure in heart cavities and large blood vessels might be estimated on the basis of the regressing property of microbubble’s subharmonic amplitude to ambient pressure. However, the pressure sensitivity is too low to satisfy clinical diagnostics requirements. Generally, a pressure measurement accuracy of 3 mmHg is desirable in medical practice. Therefore, it is still necessary to improve the ambient pressure sensitivity of the approach. In this study, we experimentally demonstrated that the ambient pressure sensitivities of first harmonic (*f*), subharmonic (1/2*f*) and ultraharmonic (3/2*f*) amplitudes from microbubbles could be improved significantly by tuning the mechanical index ($\mathrm{MI}=P_{A}/\sqrt{f}$) defined as the ratio of the acoustic pressure $P_{A}$ (MPa) to the square root of the driving frequency *f* (MHz). 

## 2. Materials and Methods

### 2.1. Ultrasound Contrast Agent Microbubbles

**Figure 1:**
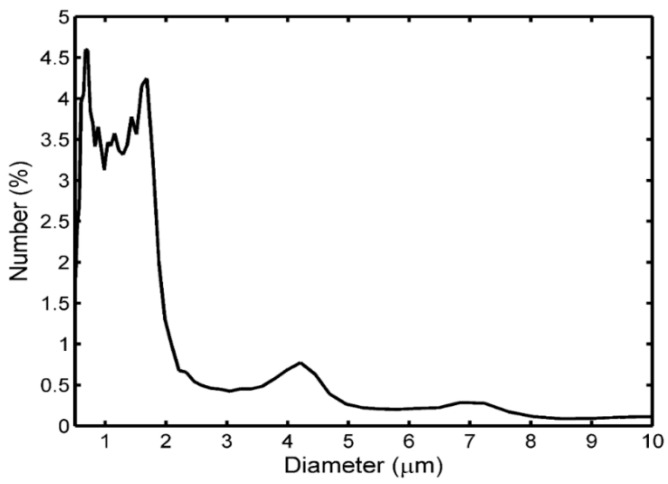
Size distribution of SonoVue microbubbles. 95% of microbubbles were in the diameter range of 0.7–10 μm and the mean diameter of the range was 2.5 μm.

Commercial UCA microbubbles, SonoVue (Bracco, Milan, Italy), were used in this study. The SonoVue contained sulfurhexafluoride free gas with phospholipid shells. By the method of hand shake, the SonoVue microbubble suspension was prepared after injecting 5 mL 0.9% NaCl solution into a septum-sealed vial with SF_6_ and 25 mg lyophilized product. After preparation, the size distribution of SonoVue microbubbles was immediately measured by laser light obscuration and scattering (Accusizer 780A, NICOMP Particle Sizing Systems, Santa Barbara, CA, USA). The resulting microbubbles with a mean diameter of 2.5 μm had size distribution approximately from 0.7 μm to 10 μm. About 95% of these bubbles were smaller than 10 μm (Figure 1).

### 2.2. Acoustic Attenuation Measurement

**Figure 2:**
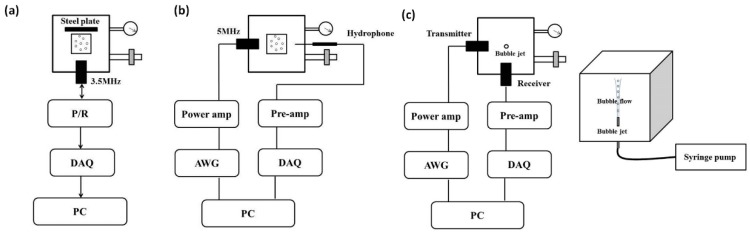
Schemes of the experimental setups for measurements of microbubbles’ acoustic properties in an ambient pressure range of 4–180 mmHg and at different time (<35 min) after injecting microbubbles. (**a**) Attenuation measurements; (**b**) Acoustic transmission measurement; (**c**) Scattering measurements and the bubble flow injection system in the 8−180 mmHg ambient pressure range. For the setup, (**a**) the Pulser/Receiver (P/R) generated and received pulses. For the setup, (**b**) the arbitrary waveform generator (AWG) connected to the power amplifier (Power amp) generated tone bursts and the transmitted signals from microbubbles were received, amplified, and digitalized by the data acquisition card (DAQ). Furthermore, the signal processing was executed by a personal computer. For (**a**) and (**b**), ultrasound contrast agents (UCA) microbubbles were added into a sample chamber. For the setup, (**c**) the diluted SonoVue microbubbles were pumped into the water tank by a syringe pump, and the bubble flow was located at the focus of the transducers. The ambient pressure was controlled by an air pump and monitored by a pressure sensor.

Figure 2a demonstrates the experimental setup to measure ultrasonic attenuation based on the broadband pulse method. A single element transducer had a center frequency of 3.5 MHz, with a −6 dB bandwidth of 2.58–5.47 MHz (V382-SU, Panametrics, Waltham, MA, USA). The transducer had an element diameter of 13 mm and a focal length of 40 mm. The transducer with the center frequency of 3.5 MHz was used as both the acoustic transmitter and receiver and the method of the acoustic attenuation measurement was the same as that described by Hoff [35].

For each measurement, the SonoVue microbubble suspension was injected into a sample chamber (length × width × height: 5 cm × 5 cm × 10 cm) which was located between the transducer and a stiff plate for reflecting acoustic waves. The chamber center was located at the focal position of the transducers. A 6-μm thick mylar acoustic window was on each side of the chamber (Goodfellow Cambridge Ltd., Cambridge, UK), allowing the passing ultrasound beam with minimal attenuation. To keep the suspension uniform, we used a magnetic stirrer at a low rotation speed in the sample chamber during measurements. A pulser/receiver transmitted a short pulse with a low energy of 12.5 μJ and a pulse repetition frequency of 1 kHz to excite the transducer. Due to the reflection of the stiff plate, the transmitted acoustic wave passed through the sample chamber twice. The received signal was amplified with a 20 dB preamplifier, and digitized at a sampling frequency of 100 Msa/s (Octopus 822F, Gage, Lockport, IL, USA). 

The measurement procedure was divided into reference and sample measurements. For the reference measurement, 200 mL 0.9% NaCl solution was added into the sample chamber. Because the transmitted acoustic wave passed through the chamber twice, the acoustic path length in the chamber is z=10 cm. The power spectrum $S_{ref}\left(f\right)$ was obtained using the fast Fourier transform (FFT) of the average of 64 received signals. And then, the sample measurement was performed after injecting 100 μL undiluted SonoVue suspensions. The power spectrum $S_{uca}\left(f\right)$ was calculated by the same method. The acoustic attenuation coefficient α(f) can be further obtained by the following formula [36].

(1) $$\alpha \left(f\right)=\frac{1}{z}8.686\left(\ln\left(S_{ref}\right)-\ln\left(S_{ref}\right)\right)\;$$

### 2.3. Acoustic Transmission Measurement

In order to determine the optimal driving frequency to generate the subharmonic scattering, we investigated the effect of the driving frequency on the subharmonic scattering power from UCA microbubbles by using the experimental setup in Figure 2b. For each measurement, 100 μL undiluted SonoVue microbubble suspension was injected into the sample chamber which contains 200 mL saline. A 5 MHz transducer with a −6 dB bandwidth of 3.3 MHz–7.33 MHz was used to transmit acoustic waves, and a needle hydrophone was used to receive the transmitted acoustic signals. A programmable arbitrary waveform generator (AFG 3102, Tektronix, Beaverton, OR, USA) generated transmitted signals, which were further amplified via a broadband radio frequency power amplifier (150A100B, Amplifier Research, Souderton, PA, USA). The transmitted signals were received by the hydrophone and were further amplified with a low noise, 40 dB preamplifier (Model 5800PR, Panametrics, Waltham, MA, USA), and digitized at a sampling frequency of 100 Msa/s. The frequencies of transmitted 64 sinusoidal bursts with a PRF of 1000 Hz acoustic waves were increased from 3 MHz to 7.4 MHz, with a frequency step of 0.2 MHz. The acoustic pressure of the focal position was 400 kPa for all the driving frequencies. The acoustic pressure was calibrated by a membrane hydrophone (HMB-0500, ONDA, Sunnyvale, CA, USA).

The received pulses by the hydrophone were further analyzed in the computer by the software Matlab 7.0 (Math-works Inc., Natick, MA, USA). For each driving frequency, the averaged subharmonic amplitude of 16 received signals was computed using FFT. To eliminate the impact of the receiver’s transfer function on the amplitude of the received signals, the subharmonic voltage amplitude was transferred to the acoustic pressure amplitude.

### 2.4. Acoustic Scattering Measurement

The acoustic scattering measurement was carried out to obtain the scattered subharmonic amplitude at different ambient pressures. The experimental system was shown in Figure 2c. Using an arbitrary waveform generator, 64 cycles of tone bursts with a PRF of 1 kHz were. There were two driving frequencies, *f*, used in the measurement, namely 4 MHz and 1.33 MHz. We used the 3.5 MHz transducer to transmit the 4 MHz acoustic wave and a 2.25 MHz transducer was used as the receiver. A 1.33 MHz acoustic wave was generated by a 1.2 MHz transducer and a 1 MHz transducer was used to receive transmitted signals. The received signals scattered by microbubbles were amplified with a low noise 40 dB preamplifier and digitized at a sampling frequency of 100 Msa/s. We used the membrane hydrophone to calibrate the acoustic pressures (350 kPa, 450 kPa, and 500 kPa) at the focus. The ambient pressure in the water tank was monitored by a pressure sensor (YZD-2B, China Academy of Aerospace Aerodynamics, Beijing, China) and manually controlled by an air pump.

We carried out the data post processing for the received pulses by using Matlab 7.0 (Math-works Inc., Natick, MA, USA). For each measurement, FFT was implemented for 50 received signals to obtain the average of their subharmonic amplitudes. For five sets of measurements, the mean values and standard deviations were computed at each ambient pressure.

For each acoustic scattering measurement, native SonoVue microbubble suspension was diluted 1000 times, and we used a syringe pump (33 Twin Syringe Pump, Harvard Apparatus, Holliston, MA, USA) to pump 10 mL diluted suspension into the water tank at a flow rate of 5 mL/min. Fresh bubbles continuously passed through the focus of the transducers under the bubble flow injection system. After each measurement, the fresh gas saturated water was poured into the tank to replace the previous water containing microbubbles.

## 3. Results

### 3.1. The Resonance Frequency of UCA Microbubbles 

**Figure 3:**
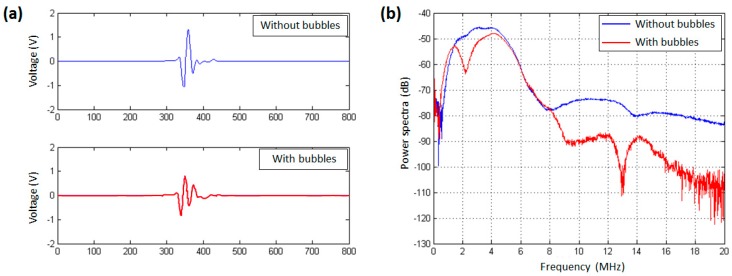
Received pulses obtained using a pair of focused transducers with a center frequency of 3.5 MHz. (**a**) Measured pulses reflected with a stainless-steel plate behind the sample chamber. The upper pulse comes from the reference measurement in the sample chamber without bubbles; the lower one is received from the transmitted pulse through the chamber with microbubbles. (**b**) The power spectra of the two received pulses.

The received signals from the reference measurement and sample measurement are shown in Figure 3a. The transmitted pulses reflected by the stiff plate passed through the sample chamber twice. From the Figure 3b, there was an obvious dip in the power spectrum of microbubble suspensions at the frequency of about 2 MHz. There was a distinct separation around 2 MHz between the spectrum from the sample without microbubbles and the spectrum from the one with microbubbles. This result indicated that the acoustic attenuation of microbubbles reached a maximum value near 2 MHz frequency corresponding to the resonance frequency of microbubbles.

**Figure 4:**
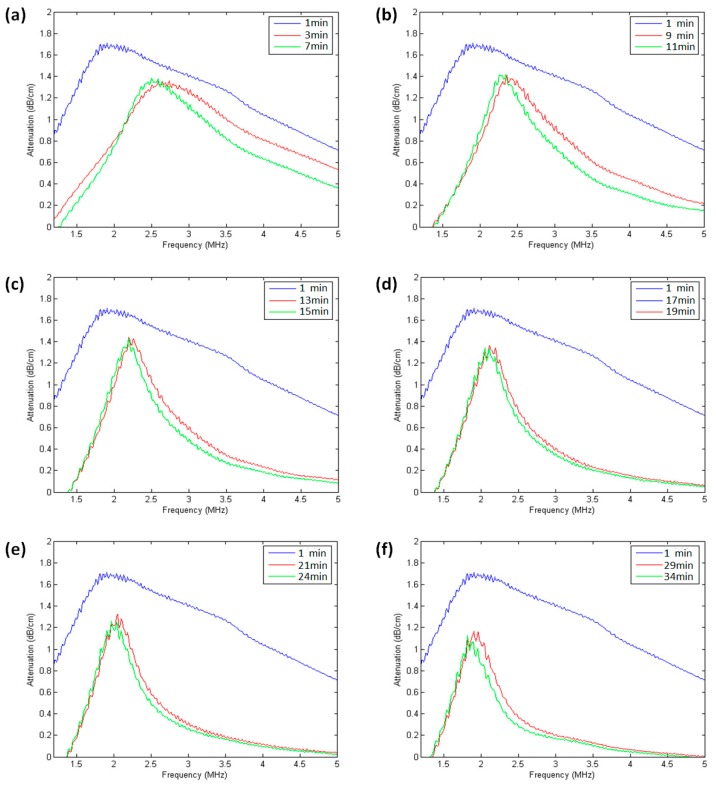
Acoustic attenuation spectra at different time after injecting SonoVue microbubbles suspensions. (**a**) 1, 3, and 7 min, (**b**) 1, 9, and 11 min, (**c**) 1, 13, and 15 min, (**d**) 1, 17, and 19 min, (**e**) 1, 21, and 24 min, and (**f**) 1, 29, and 34 min after injection.

**Figure 5:**
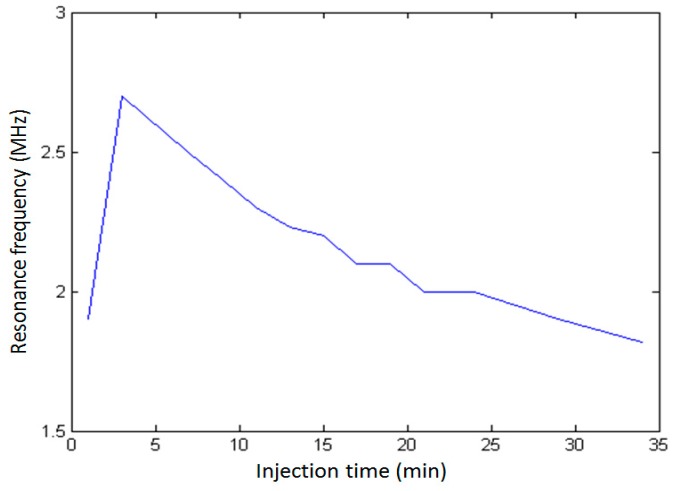
The UCA resonance frequency as a function of time from UCA injection.

In order to investigate the effect of UCA injection time on its resonance frequency, 100 μl undiluted SonoVue suspension was added into the sample chamber with 200 mL saline and measured the attenuation spectra of the same SonoVue microbubble suspension at a different time after injection. As shown in Figure 4, the maximum value of the attenuation coefficient occurred around 1.9 MHz at 1 min after bubble injection. After 3 min, the resonance frequency was increased to 2.7 MHz; after 7 min, it decreased to 2.5 MHz, with a narrower resonance peak than those before 7 min. Figure 5 showed the relationship between UCA resonance frequency and injection time. From 1 min to 3 min, the resonance frequency increased from 1.9 MHz to 2.7 MHz; after 34 min, the resonance frequency was decreased from 2.7 MHz to 1.82 MHz. The results indicated that UCA injection time had a significant effect on its resonance frequency. The detailed discussions will be given later.

### 3.2. Effects of Driving Frequency on Subharmonic Scattering Power

**Figure 6:**
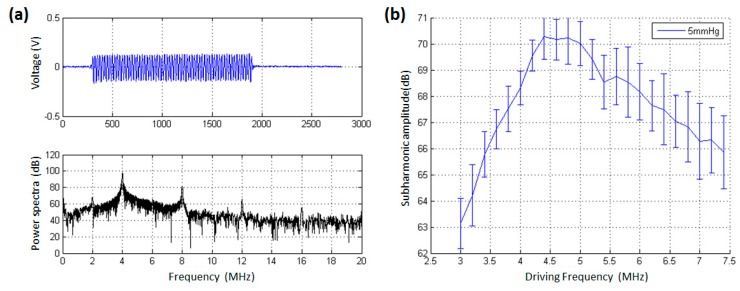
The effect of driving frequency on the subharmonic scattering power from SonoVue microbubbles at 9 min after injecting them. (**a**) An example of the received scattering signals from microbubbles and the corresponding power spectrum. A 64 cycles tone burst was transmitted at the driving frequency of 4 MHz. (**b**) The subharmonic scattering power as a function of driving frequency. The overpressure was 5 mmHg.

**Figure 7:**
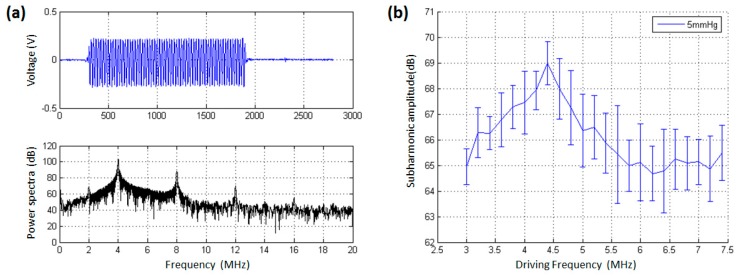
The effect of driving frequency on the subharmonic scattering power from SonoVue microbubbles at 13 min after injecting them. (**a**) An example of the received scattering signals from microbubbles and the corresponding power spectrum. A 64 cycles tone burst was transmitted at the driving frequency of 4 MHz. (**b**) The subharmonic scattering power as a function of driving frequency. The overpressure was 5 mmHg.

On the basis of measured resonance frequency, we further determined the optimal driving frequency to generate subharmonic scattering. Figure 6 and Figure 7 demonstrated the received signals after microbubble injection and the relationship between the subharmonic scattering power and the driving frequency. As shown in Figure 6a, at 9 min after bubble injection, there was also obvious subharmonic (2 MHz) amplitude, the fundamental harmonic (4 MHz), second harmonic (8 MHz), and third harmonic (12 MHz) in the power spectra. It was noted that the received acoustic transmission signals contained forward scattering signals from microbubbles and transmitted acoustic wave from the transducer. Therefore, the second harmonic, third harmonic, and fourth harmonic components partly came from the nonlinear propagation of transmitted acoustic wave, while all the subharmonic component resulted from the microbubbles’ nonlinear oscillations. As shown in Figure 6b, the subharmonic scattering power had a maximum value in the frequency range of 4.4–4.8 MHz. From 4.4 MHz to 3.0 MHz, the subharmonic power decreased by 7.13 dB, and its amplitude had a reduction of 4.37 dB in the frequency range of 4.8–7.4 MHz. Obviously, the UCA’s resonance frequency was about 2.4 MHz at 9 min after injection, as shown in Figure 4b. It was theoretically and experimentally validated that the acoustic pressure threshold to generate subharmonic scattering for a microbubble had a minimum value at the driving frequency, twice the microbubble’s resonance frequency [9,10,37,38]. In other words, there was an optimal driving frequency for subharmonic scattering, at which the subharmonic scattering power reached a maximum value under the same incident acoustic pressure. Therefore, the optimal driving frequency for subharmonic scattering should be 4.8 MHz at 9 min after bubble injection.

As shown in Figure 7a, at 13 min after bubble injection, the voltage of received signals was higher than the amplitude level at 9 min after bubble injection. The reason may attribute that more and more microbubbles were broken and levitated in the top layer, leading to the microbubble number decrease in the acoustic exposure region. In this context, it will allow much more acoustic energy to pass through the sample chamber. In addition, the microbubble numbers reduction also generated weak scattering signals, leading to a low signal to noise ratio. The resonance frequency was 2.23 MHz at 13 min after injection, as shown in Figure 4c, and the optimal driving frequency of subharmonic scattering occurred at the driving frequency of 4.4 MHz (Figure 7b), close to the predicted 4.46 MHz according to the twice resonance frequency theory [37,38]. 

### 3.3. The Relationship between Scattering Power and Ambient Pressure

#### 3.3.1. Acoustic Scattering Signals at 0.175 MI

**Figure 8:**
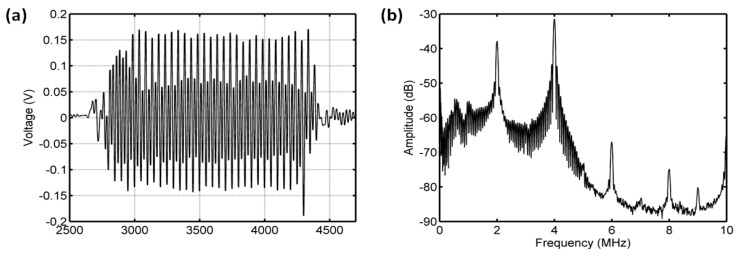
The measured scattering signals from microbubbles and the averaged power spectrum of 50 received signals. (**a**) A single received signal, (**b**) the averaged power spectrum. The driving frequency was 4 MHz, the acoustic pressure was 350 kPa, and the overpressure was 8 mmHg.

**Figure 9:**
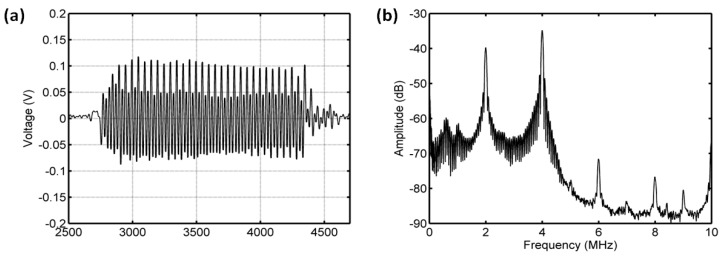
The measured scattering signals from microbubbles and the averaged power spectrum of 50 received signals. (**a**) A single received signal, (**b**) the averaged power spectrum. The driving frequency was 4 MHz, the acoustic pressure was 350 kPa, and the overpressure was 180 mmHg.

**Figure 10:**
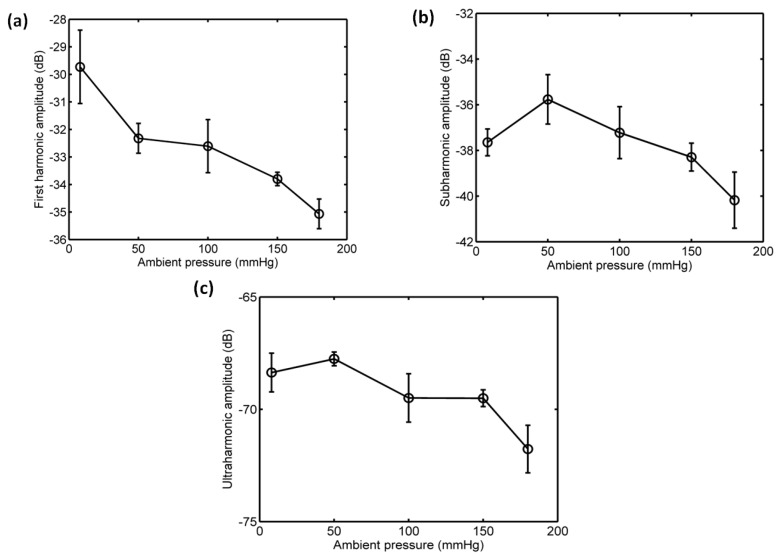
The relationships between the frequency components of the microbubbles’ scattered signals and the overpressure. (**a**) The first harmonic amplitude vs. ambient pressure, (**b**) the subharmonic amplitude vs. ambient pressure, and (**c**) the ultraharmonic amplitude vs. ambient pressure. The driving frequency was 4 MHz, the acoustic pressure was 350 kPa. The transmitted tone burst with 64 cycles had a PRF of 1 kHz.

The average of 50 received signals and the corresponding power spectrum at ambient pressures of 8 mmHg and 180 mmHg are shown in Figure 8 and Figure 9. Obviously, under the driving frequency 4 MHz and acoustic pressure 350 kPa (MI = 0.175), there were subharmonic (2 MHz), fundamental harmonic (4 MHz), ultraharmonic (6 MHz), and second harmonic (8 MHz) signals in the power spectra. Although the amplitudes of received signals significantly decreased with the increase of ambient pressure from 8 mmHg to 180 mmHg, the subharmonic amplitude was only reduced by 1.87 dB, and the fundamental and second harmonic amplitudes had reductions of 3.36 dB and 4.56 dB, respectively. The relationships between the fundamental, subharmonic, and ultraharmonic amplitudes and the ambient pressure are shown in Figure 10. The relationship between the fundamental amplitude and ambient pressure had a liner correlation coefficient of 0.9198 and ambient pressure sensitivity of −32.21 mmHg/dB; for the subharmonic case, the liner correlation coefficient and pressure sensitivity were 0.5322 and −67.98 mmHg/dB; the liner correlation coefficient and pressure sensitivity were 0.7437 and −50.44 mmHg/dB for the ultraharmonic amplitude. 

#### 3.3.2. Acoustic Scattering Signals at 0.225 MI

**Figure 11:**
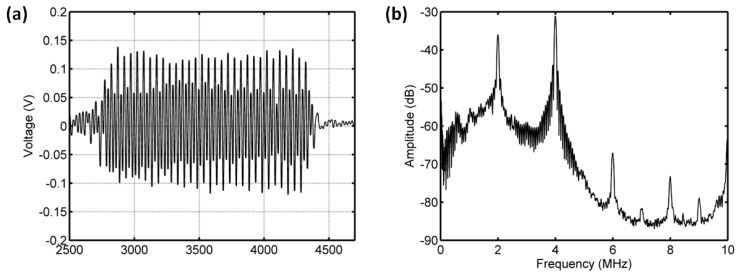
The measured scattering signals from microbubbles and the averaged power spectrum of 50 received signals. (**a**) A single received signal, (**b**) the averaged power spectrum. The driving frequency was 4 MHz, the acoustic pressure was 450 kPa, and the overpressure was 8 mmHg.

**Figure 12:**
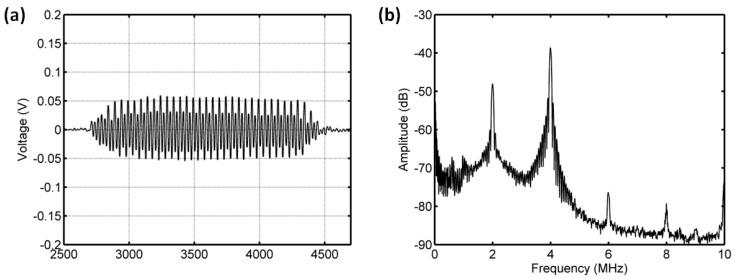
The measured scattering signals from microbubbles and the averaged power spectrum of 50 received signals. (**a**) A single received signal, (**b**) the averaged power spectrum. The driving frequency was 4 MHz, the acoustic pressure was 450 kPa, and the overpressure was 180 mmHg.

**Figure 13:**
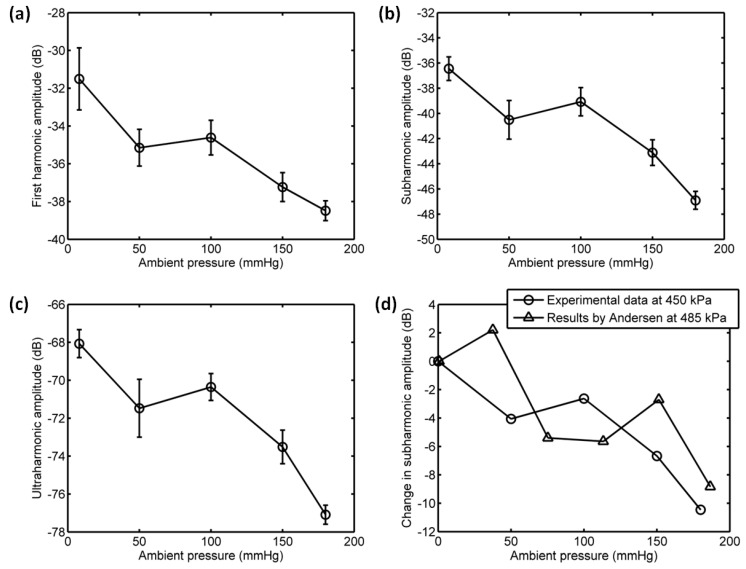
The relationships between the frequency components of the microbubbles’ scattered signals and the overpressure. (**a**) The first harmonic amplitude vs. ambient pressure, (**b**) the subharmonic amplitude vs. ambient pressure, (**c**) the ultraharmonic amplitude vs. ambient pressure, and (**d**) the comparison between the measurement in this study and the reported experimental results. The driving frequency was 4 MHz, the acoustic pressure was 450 kPa. The transmitted tone burst with 64 cycles had a PRF of 1 kHz.

As shown in Figure 11 and Figure 12, for the driving frequency 4 MHz and acoustic pressure 450 kPa, the subharmonic amplitude had an obvious decrease of 11.85 dB, and the fundamental and ultraharmonic amplitudes had reductions of 9.34 dB and 7.53 dB. The relationship between the fundamental amplitude and ambient pressure had a liner correlation coefficient of 0.8825 and ambient pressure sensitivity of −24.68 mmHg/dB; for the subharmonic case, the linear correlation coefficient and pressure sensitivity were 0.8285 and −16.44 mmHg/dB; the liner correlation coefficient and pressure sensitivity were 0.8232 and −19.07 mmHg/dB for the ultraharmonic amplitude (Figure 13). The reported subharmonic amplitude had a reduction of 9.2 dB and an ambient pressure sensitivity of −20.44 mmHg/dB, and the relationship between the subharmonic amplitude and ambient pressure had a linear correlation coefficient of 0.94. Our measurements (see Figure 13) were in agreement with the results by Andersen et al [3].

#### 3.3.3. Acoustic Scattering Signals at 0.25 MI

**Figure 14:**
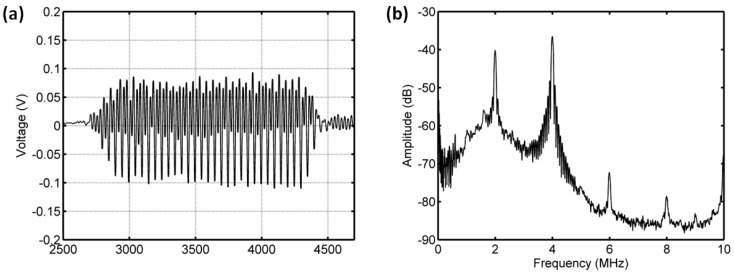
The measured scattering signals from microbubbles and the averaged power spectrum of 50 received signals. (**a**) A single received signal, (**b**) the averaged power spectrum. The driving frequency was 4 MHz, the acoustic pressure was 500 kPa, and the overpressure was 8 mmHg.

**Figure 15:**
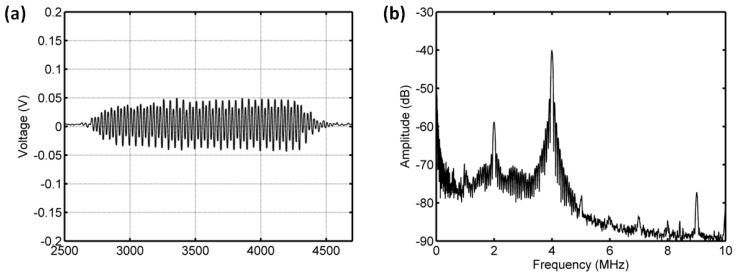
The measured scattering signals from microbubbles and the averaged power spectrum of 50 received signals. (**a**) A single received signal, (**b**) the averaged power spectrum. The driving frequency was 4 MHz, the acoustic pressure was 500 kPa, and the overpressure was 180 mmHg.

**Figure 16:**
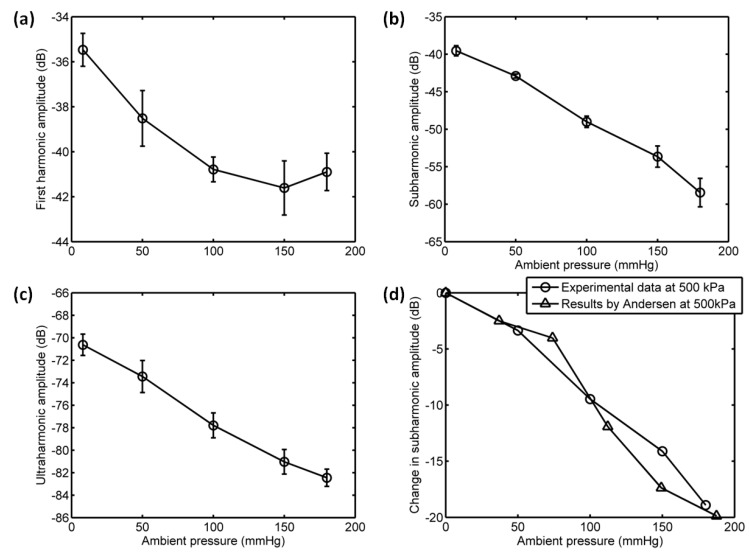
The relationships between the frequency components of the microbubbles’ scattered signals and the overpressure. (**a**) The first harmonic amplitude vs. ambient pressure, (**b**) the subharmonic amplitude vs. ambient pressure, (**c**) the ultraharmonic amplitude vs. ambient pressure, and (**d**) the comparison between the measurement in this study and the reported experimental results. The driving frequency was 4 MHz, the acoustic pressure was 500 kPa. The transmitted tone burst with 64 cycles had a PRF of 1 kHz.

Interestingly, the subharmonic amplitude had a significant reduction, achieving 18.44 dB, and the fundamental and ultraharmonic amplitudes had decreases of 3.5 dB and 11.85 dB for the driving frequency of 4 MHz and acoustic pressure of 500 kPa (Figure 14 and Figure 15). The relationship between the fundamental amplitude and ambient pressure had a liner correlation coefficient of 0.809 and ambient pressure sensitivity of −31.68 mmHg/dB; for the subharmonic case, the liner correlation coefficient and pressure sensitivity were 0.9919 and −9.1 mmHg/dB; the liner correlation coefficient and pressure sensitivity were 0.9934 and −14.55 mmHg/dB for the ultraharmonic amplitude (Figure 16). Our measurements agreed very well with the reported results [3]. In their study, the subharmonic amplitude had a reduction of 19.75 dB, an ambient pressure sensitivity of −9.52 mmHg/dB, and a linear correlation coefficient of 0.9581 between the subharmonic amplitude and ambient pressure.

#### 3.3.4. Acoustic Scattering Signals at 0.3 MI

**Figure 17:**
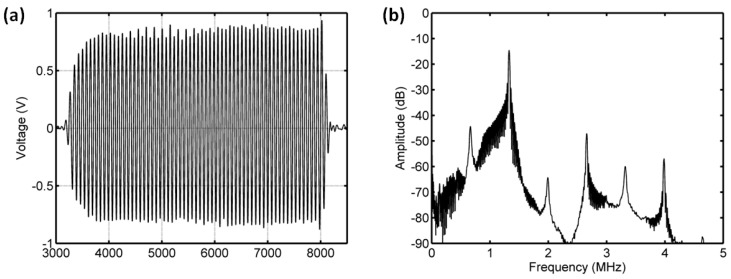
The measured scattering signals from microbubbles and the averaged power spectrum of 50 received signals. (**a**) A single received signal, (**b**) the averaged power spectrum. The driving frequency was 1.33 MHz, the acoustic pressure was 350 kPa, and the overpressure was 8 mmHg.

**Figure 18:**
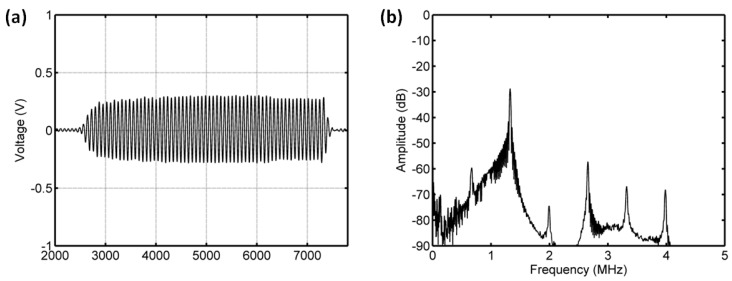
The measured scattering signals from microbubbles and the averaged power spectrum of 50 received signals. (**a**) A single received signal, (**b**) the averaged power spectrum. The driving frequency was 1.33 MHz, the acoustic pressure was 350 kPa, and the overpressure was 180 mmHg.

**Figure 19:**
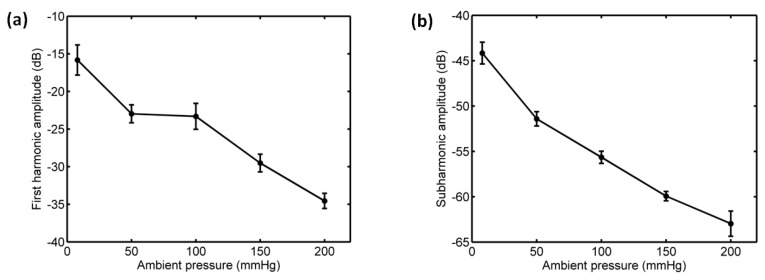

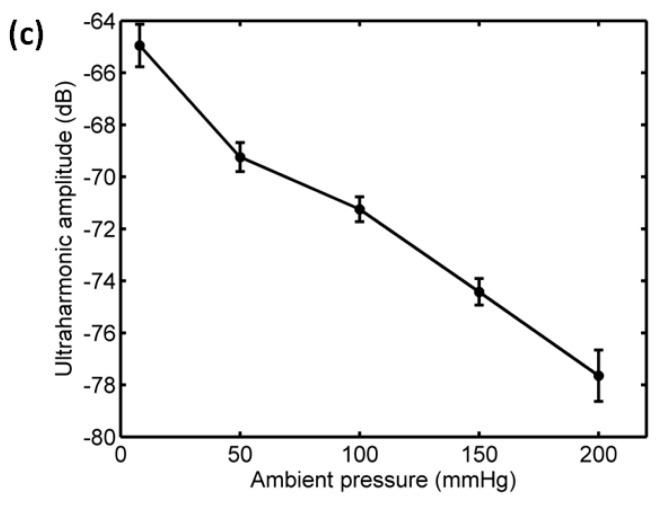
The relationships between the frequency components of the microbubbles’ scattered signals and the overpressure. (**a**) The first harmonic amplitude vs. ambient pressure, (**b**) the subharmonic amplitude vs. ambient pressure, and (**c**) the ultraharmonic amplitude vs. ambient pressure. The driving frequency was 1.33 MHz, the acoustic pressure was 350 kPa. The transmitted tone burst with 64 cycles had a PRF of 1 kHz.

Furthermore, we also detected the acoustic scattering signals at 0.3 MI at the driving frequency 1.33 MHz and acoustic pressure 350 kPa. We found the subharmonic amplitude had a reduction of 15.26 dB and the fundamental and ultraharmonic amplitudes had decreases of 14.22 dB and 10.15 dB (Figure 17 and Figure 18). The relationship between the fundamental amplitude and ambient pressure had a liner correlation coefficient of 0.9503 and ambient pressure sensitivity of −10.25 mmHg/dB; for the subharmonic case, the liner correlation coefficient and pressure sensitivity were 0.9633 and −10.21 mmHg/dB; the liner correlation coefficient and pressure sensitivity were 0.9846 and −15.12 mmHg/dB for the ultraharmonic amplitude (Figure 19).

Table 1, Table 2 and Table 3 summarize the ambient pressure sensitivity of the first harmonic, subharmonic, and ultraharmonic amplitudes at different acoustic parameters during 0–180 mmHg, respectively. The reduction of the first harmonic amplitude reached a value of 18.53 dB at 1.33 MHz, much higher than those values at 4 MHz (Table 1). It is noted that there was a maximum reduction of the first harmonic amplitude for the case of 4 MHz, along with the increase of acoustic pressure from 350 kPa to 450 kPa, and then decreasing with the acoustic pressure. The reduction of the ultraharmonic amplitude became apparently as MI increased (Table 3). Especially, a maximum value of 12.55 dB reduction was obtained at the driving frequency 1.33 MHz and acoustic pressure 350 kPa. 

At the driving frequency 4 MHz, the reduction of the subharmonic amplitude, ambient pressure sensitivity, and the linear correlation between the ambient pressure and subharmonic amplitude had been significantly improved as the acoustic pressure and MI increase, as shown in Table 2. For the acoustic pressure of 350 kPa, MI also can be raised when the driving frequency was reduced from 4 MHz to 1.33 MHz. Similarly, the reduction of subharmonic amplitude, ambient pressure sensitivity, and the linear correlation between the ambient pressure and subharmonic amplitude had also improved. Notably, the subharmonic amplitude was increased by 81.7% and 61.5% at mechanical indexes of 0.25 and 0.3 in comparison with a MI = 0.225. Thus, it was indicated that increasing the mechanical index MI can improve the subharmonic amplitude’s ambient pressure sensitivity.

## 4. Discussion

Microbubbles have been widely applied in clinical diagnoses. Thanks to their excellent scattering properties, contrast to tissue ratio of images can be greatly improved. In spite of the medical imaging application, the scattering echo signals as a function of ambient pressure and makes microbubbles as potential pressure sensors for noninvasive blood pressure estimation. In this study, we demonstrated the effect of UCA injection time on the resonance frequency and also found that the mechanical index had a distinct impact on the ambient pressure sensitivity of the scattering power. 

**Figure 20:**
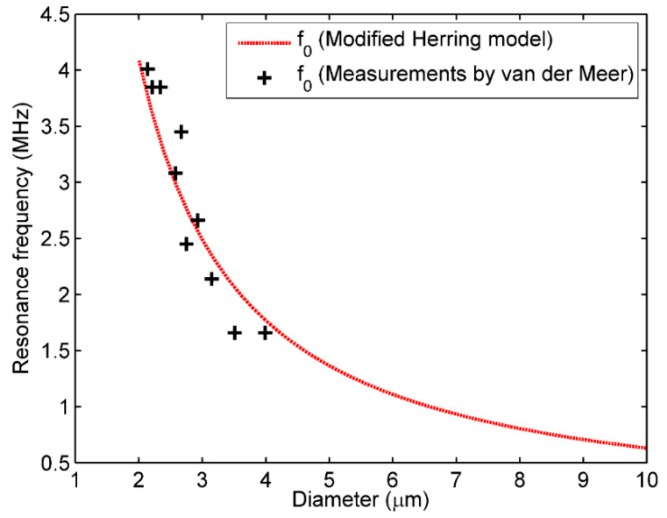
The resonance frequency of a SonoVue microbubble as a function of its diameter.

Our data indicated that UCA injection time had a significant effect on its resonance frequency (Figure 4 and Figure 5). According to the calculated resonance frequencies derived from the modified Herring model and the measurements for SonoVue microbubbles [39], the resonance frequency was increased with the decreasing microbubble’s size (Figure 20). Therefore, it is reasonable to explain that the resonance frequency as a function of injection time may be caused by the microbubble size distribution variation generated by the microbubble’s destruction. Chomas et al. studied the destruction mechanism of UCA MP1950 microbubbles and revealed the destruction mechanisms of these lipid microbubbles can be divided into fragmentation, acoustically driven diffusion, and static diffusion [40]. The acoustically driven diffusion was responsible for the inner gas diffusion into the surrounding liquid under the acoustic excitation. This type of destruction process lasted tens of milliseconds and there was no static diffusion occurring during non-exposure period. A MP1950 microbubble’s size decrease did not stop until the acoustic exposure was turned off. Further experimental results demonstrated that the resonance frequency was increased with the microbubbles’ size reduction generated by the acoustically driven diffusion [40]. However, it is also notable that the microbubble size will not always be reduced under the acoustic exposure. Borden et al. found that there was a stable equilibrium diameter for a lipid-coated microbubble [41]. When a microbubble’s initial equilibrium diameter was larger than the stable value, the microbubble’s size will be decreased to the stable value under the acoustically driven diffusion. In addition, it was not observed that the microbubble’s size was changed when the initial diameter was smaller or equal to the static value. 

The results of Figure 4 and Figure 5 can be explained as follows. Initially, the sizes of SonoVue microbubbles with initial diameters larger than a stable value were rapidly decreased to the stable diameter to increase the UCA suspension’s resonance frequency, due to the effect of acoustically driven diffusion. After a long enough exposure time, a large number of microbubbles with initial diameters smaller than the stable size were broken, thus decreasing the UCA suspension’s resonance frequency. In addition, for initial SonoVue suspension with an original size distribution in Figure 1, most of the acoustic scattering power was provided by microbubbles in the 3 to 9 μm diameter range [42], and these microbubbles had a 0.7–2.5 MHz resonance frequency range. Therefore, the resonance frequency had a low starting value of 1.9 MHz at 1 minute after injecting microbubbles.

The subharmonic scattering amplitude was dependent on the acoustic pressure. It was believed that there were three acoustic pressure stages for the generation of subharmonic scattering signals, namely occurrence, growth, and saturation [1]. At the growth stage, the subharmonic signals from microbubbles had a high amplitude reduction with the ambient pressure variation and can be used to estimate the blood pressure. It was reported that the growth stage of subharmonic signals from SonoVue microbubbles appeared in the acoustic pressure range of 0.3 MPa–0.5 MPa [3]. At the initial growth stage, the ambient pressure usually had a little impact on the subharmonic amplitude and a low ambient pressure sensitivity was obtained at this stage. Therefore, only a low reduction of 2.53 dB was observed in our study at the initial growth stage (350 kPa). In contrast, at the end of growth stage, a higher subharmonic amplitude reduction can be obtained to accurately estimate the ambient pressure. Therefore, we obtained a subharmonic amplitude reduction of 10.9 dB at the growth end (450 kPa).

It is noted that there was an excellent linear correlation between the subharmonic amplitude and ambient pressure at the acoustic pressure of 500 kPa, and a significant amplitude reduction reached a value of 18.91 dB when the ambient pressure was increased from 8 mmHg to 180 mmHg. Our measurement agreed with the observation by Andersen et al [3] and it was very different from the previous reports that most UCAs such as Levovist, Optison, Definity, PRC-1, and Sonazoid had a subharmonic amplitude reduction of 9–12 dB [20,24]. This high amplitude reduction may be caused by the destruction of microbubbles. The acoustically driven diffusion for lipid-coated microbubbles occurred in the acoustic pressure range of 400–600 kPa, while a higher acoustic pressure (800 kPa) was usually needed to induce a fragmentation of a microbubble. Therefore, the destruction mechanism of microbubbles at an acoustic pressure of 500 kPa was dominated by the acoustically driven diffusion. On the other hand, the disappearance time (dissolve rate) of a free bubble was related to the ambient pressure. The higher the ambient pressure was, the faster the gas dissolved, and the more destroyed microbubbles were [5]. Thus, an increase of ambient pressure also accelerated the rate of acoustically driven diffusion, resulting in the decrease of microbubbles’ concentration. Because the ambient pressure was proportional to the number of destroyed microbubbles, there was an excellent linear correlation between the ambient pressure and subharmonic amplitude. Obviously, the subharmonic amplitude reduction caused by the decrease of microbubble concentration was higher than that of UCA suspensions with unbroken microbubbles.

In general, there were two ways to increase MI to destroy microbubbles, namely increasing the acoustic pressure and decreasing the driving frequency. In order to further validate the microbubble destruction induced high reduction of subharmonic amplitude, the relationship between the subharmonic amplitude and ambient pressure was obtained at the driving frequency of 1.33 MHz and acoustic pressure of 350 kPa. The subharmonic amplitude reduction and linear correlation coefficient were in accordance with the results at 4 MHz and 500 kPa. Mannaris et al. investigated the effect of duration time (10–20,000 cycles) of long pulses on SonoVue microbubbles’ oscillations [43]. Few microbubbles were destroyed and the scattering power almost kept a constant at MI < 0.1; at MI = 0.2, the scattering power decreased with the time, and the gas inside a microbubble was dissolved into the surrounding medium; at MI > 0.4, the scattering power was very weak after 100 cycles, indicating rapid and complete destructions of a large number of microbubbles. The mechanical index in our study was equal to 0.3. At the moderate MI, a large number of microbubbles will not be destroyed in a short time, thus there was a long enough time for the ambient pressure to affect microbubbles with gas diffusion.

The fundamental harmonic and ultraharmonic components also demonstrated significant amplitude reductions and had good liner correlations with the ambient pressure at the acoustic pressure of 350 kPa and the driving frequency of 1.33 MHz. This frequency was two thirds of the resonance frequency and it was the optimal driving frequency to generate ultraharmonic scattering signals [23]. When a microbubble’s size was decreased due to the gas diffusion caused by the ultrasonic excitation and ambient pressure, according to the linearized Hoff model [44], the microbubble’s resonance frequency will increase with the decreased radius and increased ambient pressure. As a result, the resonance frequency will deviate from the initial optimal driving frequency and the ultraharmonic scattering power will significantly decrease. It was noted that the fundamental harmonic amplitude had a reduction of 18.53 dB, which was much larger than the reported value (~2 dB) for most UCAs [1]. The reason may originate from a large number of microbubbles’ destructions caused by ambient-pressure-enhanced acoustically driven diffusion. An accurate first harmonic-aided pressure estimation method may be developed, which can make use of traditional B-mode ultrasound imaging system and can be easily integrated into current commercial scanners, without changing their system framework.

## 5. Conclusions

We experimentally investigated the effects of acoustic pressure and driving frequency on ambient pressure sensitivities of first harmonic, subharmonic, and ultraharmonic scattering powers from SonoVue microbubbles. The mechanical index had a significant effect on the correlation between the scattering power and ambient pressure. It was indicated that microbubbles’ destructions caused by the acoustically driven diffusion at a moderate MI (0.25–0.4) could significantly improve both the ambient pressure sensitivities of first harmonic, subharmonic, and ultraharmonic scattering powers and the linear correlation between the scattering power and ambient pressure by either increasing the acoustic pressure or decreasing the driving frequency. In the future, a further study will be carried out to investigate the effects of flow rate, microbubbles’ concentrations, and the variation rate of ambient pressure on the accuracy of pressure estimation in a circulation system.

## Figures and Tables

**Table 1:** Ambient pressure sensitivity of the first harmonic amplitude.

*P*a (kPa)	*f* (MHz)	MI	First harmonic (dB)	Sensitivity (mmHg/dB)	Correlation
**350**	4	0.175	5.0	−36	0.9198
**450**	4	0.225	6.57	−27.4	0.8825
**500**	4	0.25	4.58	−39.3	0.809
**350**	1.33	0.30	18.53	−9.71	0.9503

**Table 2:** Ambient pressure sensitivity of the subharmonic amplitude.

*P*a (kPa)	*f* (MHz)	MI	Subharmonic (dB)	Sensitivity (mmHg/dB)	Correlation
**350**	4	0.175	2.6	−67.98	0.5322
**450**	4	0.225	10.9	−16.44	0.8285
**500**	4	0.25	19.8	−9.1	0.9919
**350**	1.33	0.30	17.6	−10.21	0.9633

**Table 3:** Ambient pressure sensitivity of the ultraharmonic amplitude.

*P*a (kPa)	*f* (MHz)	MI	Ultraharmonic (dB)	Sensitivity (mmHg/dB)	Correlation
**350**	4	0.175	4.22	−50.44	0.7437
**450**	4	0.225	9.28	−19.4	0.8232
**500**	4	0.25	12.28	−14.66	0.9934
**350**	1.33	0.30	12.55	−14.34	0.9846
